# Modified Closed Coronary Transfer is a Good Alternative to the Trap-Door Method During Arterial Switch Operation: a Retrospective Propensity-Matched Comparison

**DOI:** 10.21470/1678-9741-2019-0263

**Published:** 2020

**Authors:** Mehmet Dedemoğlu, Gültekin Coşkun, Fatih Özdemir, Okan Yurdakök, Oktay Korun, Murat Çiçek, Mehmet Biçer, Filiz İzgi Coşkun, Numan Ali Aydemir, Ahmet Şaşmazel

**Affiliations:** 1 Department of Pediatric Cardiovascular Surgery, Dr. Siyami Ersek Thoracic and Cardiovascular Surgery Training and Research Hospital, Istanbul, Turkey.; 2 Department of Pediatric Cardiovascular Surgery, Mersin City Training and Research Hospital, Mersin, Turkey.; 3 Department of Pediatric Cardiovascular Surgery, Dr. Siyami Ersek Thoracic and Cardiovascular Surgery Training and Research Hospital, Istanbul, Turkey.; 4 Department of Pediatric Cardiovascular Surgery, Gazi Yaşargil Education and Research Hospital, Diyarbakır, Turkey.; 5 Department of Pediatric Cardiovascular Surgery, Health Sciences University Erzurum Region Education and Research Hospital, Erzurum, Turkey.; 6 Department of Anesthesia and Reanimation, Dr. Siyami Ersek Thoracic and Cardiovascular Surgery Training and Research Hospital, Istanbul, Turkey.

**Keywords:** Arterial Switch Operation, Coronary Arteries, Coronary Artery Reimplantation, Propensity Score, Transposition of great vessels, Heart, Echocardiography, Arterial Switch Operation

## Abstract

**Objective:**

To compare the early and long-term results of patients in whom was performed modified closed coronary transfer with the results of patients in whom was performed trap-door transfer techniques by utilizing propensity-matching analysis to provide optimal identical patient matching for the groups.

**Methods:**

From August 2015 to December 2017, 127 consecutive patients underwent arterial switch operation due to simple and complex transposition of the great arteries, with or without additional arch and complex coronary pattern, by a single surgical team included into the study. Of these, in 70 patients it was performed modified closed coronary transfer technique and in 57 patients it was performed trap-door style coronary transfer technique. The patients were divided into two groups in terms of coronary transfer method. In the final model, after propensity matching, 47 patients from each group having similar propensity score were included into the study.

**Results:**

There was no significant difference between the groups regarding patient characteristics. Cross-clamp time and operation time were significantly lower in the modified technique group compared with the other group (*P*=0.03 and *P*=0.05, respectively). When compared the early and late postoperative outcomes, there was no significant difference between the groups. Postoperative echocardiographic findings were mostly similar between the groups.

**Conclusion:**

The patients in whom was performed our modified technique demonstrate overall good outcomes and the current technique ensures shorter arterial cross-clamp and operation times. It may be an alternative method to the trap-door technique for the coronary transfer during the arterial switch operation.

**Table:** 

Abbreviations, acronyms & symbols				
ASO	= Arterial switch operation		Neo-PA	= Neo-pulmonary artery
CPB	= Cardiopulmonary bypass		PS	= Propensity score
Cx	= Circumflex coronary artery		PTFE	= Polytetrafluoroethylene
DORV	= Double outlet right ventricle		R	= Right coronary artery
ECMO	= Extracorporeal membrane oxygenator		SD	= Standard deviation
ICU	= Intensive care unit		SPSS	= Statistical Package for the Social Sciences
IVS	= Intact ventricular septum		TGA	= Transposition of the great arteries
L	= Left main coronary artery		VSD	= Ventricular septal defect
LOS	= Length of stay			

## INTRODUCTION

Arterial switch operation (ASO) has become a standard surgical procedure for the treatment of transposition of the great arteries (TGA) and some forms of double outlet right ventricle (DORV) with low mortality and excellent long-term results^[1,2]^, since it was first described by Jatene et al.^[3]^. Early mortality after ASO is almost always related to coronary artery failure^[4]^. The potential risk for this complication is due to impaired coronary perfusion caused by kinking, distortion, stenosis, or compression of the coronary arteries^[5]^ and that risk is increasing in the presence of abnormal coronary artery pattern^[6,7]^. Therefore, coronary artery transfer is the crucial step during ASO^[8,9]^. In the current era, technical modifications for optimal coronary configuration during coronary reimplantation have minimized the risk related to coronary artery failure^[10]^. In this context, different coronary transfer techniques have been described for coronary reimplantation^[11]^. The most commonly used method is the trap-door coronary transfer technique, which minimized the rotation angle of the coronary artery^[12]^. Besides, the closed coronary transfer technique, which provides optimal geometric configuration for the coronary arteries, is also a method accepted in the literature^[13]^.

We have currently been performing ASO for these patients’ group in our department by using a modification of the closed coronary transfer technique for optimal coronary configuration. The purpose of this study is to compare the early and long-term results of patients in whom was performed modified closed coronary transfer with the results of patients in whom was performed trap-door transfer techniques by utilizing propensity-matching analysis to provide optimal identical patient matching for the groups.

## METHODS

The ethical approval (number 28001928-508.01) was obtained from the Scientific Research Permission Commission of our institute.

### Patients

This is a retrospective observational cohort study and it was made up of data enrolling consecutive patients who underwent ASO by a single surgical team in our department between August 2015 and December 2017. The clinical data were obtained from hospital records. The patients’ demographics and laboratory and echocardiographic findings were recorded to reveal baseline patient characteristics. Only patients undergoing ASO electively due to diagnosis of TGA with or without ventricular septal defect (VSD) and Taussig-Bing anomaly were included into the study. Patients older than three weeks, who underwent palliative and/or double switch operation, who underwent ASO by other surgical team, and who had preoperative poor hemodynamic condition were excluded from the study. Finally, a total of 127 consecutive patients were included into the study. All patients were divided into two groups in terms of coronary transfer technique performed during ASO. According to this, 70 patients in whom was performed modified close coronary transfer technique were defined as group 1 and 57 patients in whom was performed trap-door coronary transfer technique were group 2.

In order to describe the coronary pattern, Leiden convention was utilized. According to this classification, the coronary pattern was defined based on the sinus (sinuses 1 and 2) of origin of coronary arteries^[14]^. With reference to this description, coronary patterns were named as usual (1LCx2R), circumflex artery from the right coronary artery (1L2CxR), single left coronary artery (1LCxR), and single right coronary artery (2LCxR).

### Surgical Technique and Postoperative Care

**Fig. 1A:**
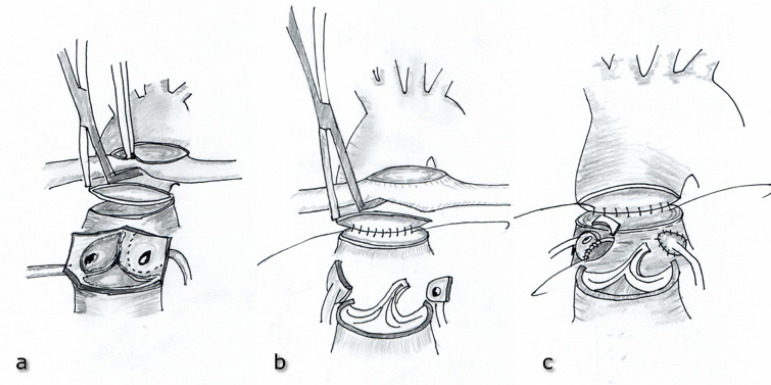
Coronary arteries are removed from their sinuses as a large ‘u shaped’ flap. **Fig. 1B** - The posterior half of anastomosis between the neo-aorta and ascending aorta is performed. **Fig. 1C** - Coronary reimplantation to the neo-aortic root.

**Fig. 2A:**
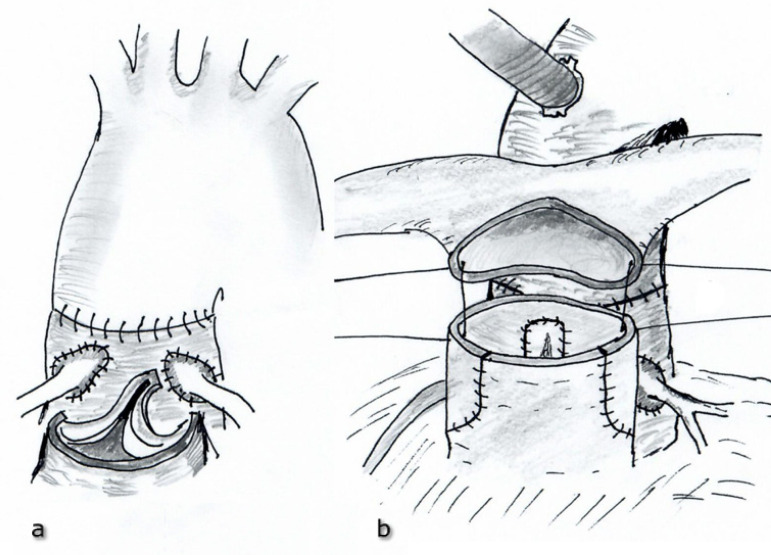
Completion of the neo-aortic reconstruction. **Fig. 2B** - Neo-pulmonary reconstruction and anastomosis between the neo-pulmonary artery and pulmonary bifurcation.

All operations were performed in a standard cardiac surgery fashion. After median sternotomy, standard aortic arterial bicaval venous cannulation was used for cardiopulmonary bypass (CPB). Myocardial protection was achieved by mild hypothermia and by antegrade infusion of del Nido cardioplegia solution into the aortic root, and then through coronary ostia, if necessary. In patients with VSD, the defect was closed by direct suturing or by using patch material (glutaraldehyde-fixed autologous pericardium) through right atrium before the transection of great arteries. VSD was also closed through neo-aorta in patients with Taussig-Bing anomaly. The wide mobilization of branch pulmonary arteries was performed in order not to occur any tension after LeCompte maneuver. After division of the patent ductus arteriosus and transection of the great arteries, coronary arteries were removed from their sinuses as a large ‘u shaped’ flap (Figure 1A). The coronary arteries were then mobilized properly to be resided on the neo-aortic root without any tension. In the modified approach, the aorta was transected 8-10 mm above the sinotubular junction, and the main pulmonary artery was transected just below its bifurcation. It was important to leave a long neo-aortic root to obtain an adequate coronary artery implantation area. After performing LeCompte maneuver, the posterior half (not all) of the anastomosis, between neo-aorta and ascending aorta, was performed, unlike the classical closed coronary transfer technique (Figure 1B). Thus, more secure control was provided to prevent injury of the neo-aortic valve during the coronary artery anastomosis. After the optimal geometric position of coronary arteries was adjusted at the neo-aortic root, incisions matching the most appropriate coronary artery alignment were made, without splitting the transected aortic rim, at these sites for the coronary implantation. Coronary arteries were then anastomosed to the neo-aortic root (Figure 1C). During coronary artery anastomosis, the aortic valve was continuously checked within the neo-aortic root to avoid neo-aortic valve injury. Following, neo-aortic reconstruction was completed (Figure 2A). In the classical approach, the coronary transfer was performed before the aortic reconstruction. The ascending aorta was transected approximately 5 mm above the sinotubular junction, and the main pulmonary artery was divided at about 2 mm proximal to its bifurcation. Open trap-door incisions were made at the site of anastomotic area and the coronary buttons were then implanted to the neo-aortic root. Thereafter, LeCompte maneuver was applied and the neo-aortic reconstruction was performed. In both approaches, the defect in the neo-pulmonary artery (neo-PA) was reconstructed using a pantaloon-shaped autologous fresh pericardial patch. The aortic cross-clamp was released, and neo-PA was then anastomosed to the pulmonary bifurcation on a beating heart (Figure 2B). The patients were weaned off CPB and the operation was completed in a standard surgical fashion. In some patients having myocardial and lung edema, sternal closure was not performed, and only skin was sutured.

After the operation, all patients were followed at the cardiac intensive care unit (ICU). Patients’ arterial blood pressure, cardiac rhythm, central venous pressure, and saturation were monitored. Inotropic support was routinely applied with low dose dopamine. If necessary, other inotropic agents were utilized. If required, peritoneal dialysis was utilized to remove high volume load.

### Outcomes and Follow-Up

The mean follow-up duration was 2.3±0.9 (min: 0.01, max: 3.8) years. All patients were followed up by routine physical examination and echocardiography periodically. Primary outcomes of interest were to compare postoperative potential outcomes, duration of ICU stay, length of hospital stay, and mortality; and to compare long-term survival rates between the groups at the follow-up.

### Propensity Score Analysis

In order to overcome the difference in terms of patient characteristics between groups and to create two identical groups with regard to potential risk factors, we performed propensity score (PS) adjustment and PS matching. As described by Blackstone^[15]^, the PSs were calculated for each patient using a non-parsimonious multivariable logistic regression model, with the group (groups 1 and 2) variable as the dependent variable and with the 12 baseline-patient parameters as the covariates. After calculating PSs for each patient, PS matching was applied at a 1:1 ratio by using of the nearest-neighbor matching. A PS difference of 0.1 was used as a maximum caliper width for matching two patients. In the final model, 47 patients from each group having similar PS were included into the study.

### Statistical Analyses

Statistical analyses were performed using the Statistical Package for the Social Sciences (SPSS) software. Parametric continuous data were expressed as mean and standard deviation, while non-parametric continuous data were expressed as median and interquartile ranges. Categorical variables were reported as frequency and percentage. Independent sample t-test for parametric data, Mann-Whitney U test for non-parametric data, and Pearson’s chi-square test for categorical data were performed for group comparisons. Survival curves were obtained with the help of Graphpad Prism Software and Long-Rank test was performed to compare survival rates of the groups. P-value ≤ 0.05 was considered to be statistically significant.

## RESULTS

**Fig. 3A:**
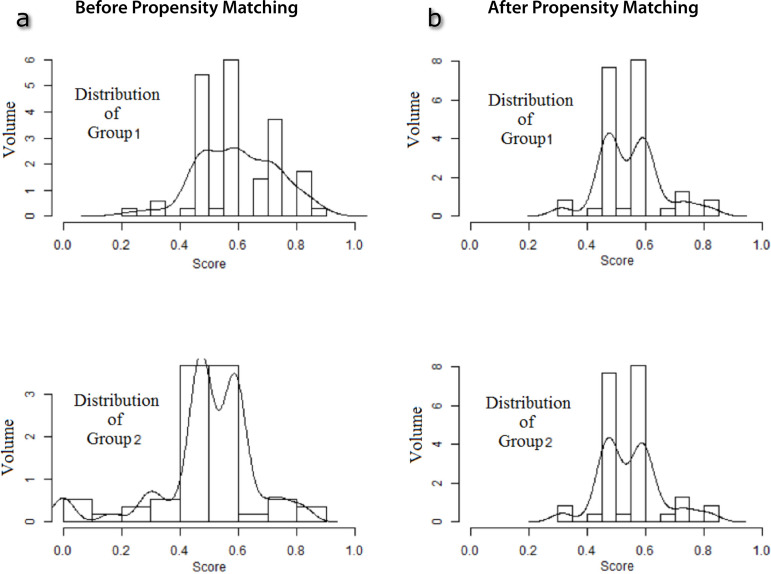
Distribution of the groups before the propensity score matching. **Fig. 3B** - Distribution of the groups after the propensity score matching.

Preoperative patient characteristics without PS matching are demonstrated in Table 1. Although there was no statistically significant difference between the two groups regarding to patient characteristics, the distribution between the groups was not identical (Figure 3A). In addition to this, there was significant difference between the groups in terms of PSs (*P*<0.001). After PS matching, the distribution between the groups was identical (Figure 3B) and there was no significant difference between the groups in terms of PSs (*P*=0.93). The final patient characteristics are listed in Table 2. Median age was 7.5 days (min: 5, max: 11) and there was male gender dominance (79.8%). Operative data and postoperative outcomes of the matched cohort are depicted in Table 3. Cross-clamp time and operation time were significantly lower in group 1 compared with group 2 (*P*=0.03 and *P*=0.05, respectively). According to other parameters in the operative findings, there was no significant difference between the groups. When compared the postoperative outcomes, there was no significant difference between the groups. One patient, who had coronary artery with intramural course in group 1, underwent reanastomosis and pericardial patch reconstruction due to the coronary perfusion problem during the operation. Mean follow-up duration was significantly lower in group 1 compared with group 2 (*P*<0.001) because the majority of patients in group 1 underwent surgical operation in the late period of the follow-up duration. Postoperative echocardiographic findings are listed in Table 4 and there was no significant difference between the groups regarding these findings.

**Table 1:** Preoperative patients' characteristics before the propensity score matching.

Variables (%, mean±SD, median-interquartile ranges)	Group 1 (n:70)	Group 2 (n:57)	All patients (n:127)	*P*-value
Age (day)	8 (5-13)	10 (6-15)	8 (5-14)	0.08
Weight at the operation (kg)	3.5±0.9	3.8±1.4	3.7±1.1	0.29
Gender				0.19
Male	48 (68.6)	45 (78.9)	93 (73.2)	
Female	22 (31.4)	12 (21.1)	34 (26.8)	
Diagnosis				
TGA-IVS	36 (51.4)	29 (50.9)	65 (51.2)	0.95
TGA-VSD	30 (42.9)	19 (33.3)	49 (38.6)	0.27
Taussig-Bing anomaly	4 (5.7)	9 (15.8)	13 (10.2)	0.06
Side-by-side relation of the great arteries	1 (1.4)	1 (1.8)	2 (1.6)	1.0
Additional anomaly				
Aortic arch hypoplasia	4 (5.7)	8 (14.0)	12 (9.4)	0.11
Interrupted aortic arch	0	3 (5.3)	3 (2.4)	0.09
Coarctation of aorta	1 (1.4)	1 (1.8)	2 (1.6)	1.0
Coronary artery pattern				1.0
Usual	64 (91.4)	52 (91.2)	116 (91.3)	
Atypical pattern	6 (8.6)	5 (8.8)	11 (8.7)	
1L2CxR	4 (5.7)	3 (5.3)	7 (5.5)	
1LR2Cx	1 (1.4)	0	1 (0.8)	
1LCxR	1 (1.4)	1 (1.8)	2 (1.6)	
2LCxR	0	1 (1.8)	1 (0.8)	
Intramural course	3 (4.3)	1 (1.8)	4 (3.1)	0.63
Preoperative septostomy	11 (15.7)	3 (5.3)	14 (11.0)	0.06
Propensity score	0.6±0.1	0.5±0.2	0.6±0.2	<0.001*

Cx=circumflex coronary artery; IVS=intact ventricular septum; L=left main coronary artery; R=right coronary artery; SD=standard deviation; TGA=transposition of the great arteries; VSD=ventricular septal defect Group 1=patients undergoing modified closed coronary transfer; Group 2=patients undergoing trap-door coronary transfer

* Statistically significant parameter

**Table 2:** Preoperative patients' characteristics after the propensity score matching.

Variables (%, mean±SD, median-interquartile ranges)	Group 1 (n:47)	Group 2 (n:47)	All patients (n:94)	*P*-value
Age (day)	7 (4-11)	9 (6-11)	7.5 (5-11)	0.35
Weight at the operation (kg)	3.4±0.5	3.4±0.4	3.4±0.4	0.84
Gender				0.80
Male	38 (80.9)	37 (78.7)	75 (79.8)	
Female	9 (19.1)	10 (21.3)	19 (20.2)	
Diagnosis				
TGA-IVS	31 (66.0)	28 (59.6)	59 (62.8)	0.52
TGA-VSD	13 (27.7)	16 (34.0)	29 (30.9)	0.50
Taussig-Bing anomaly	3 (6.4)	3 (6.4)	6 (6.4)	1.0
Side-by-side relation of the great arteries	1 (2.1)	1 (2.1)	2 (2.1)	1.0
Additional anomaly				
Aortic arch hypoplasia	3 (6.4)	3 (6.4)	6 (6.4)	1.0
Interrupted aortic arch	0	0	0	
Coarctation of aorta	1 (2.1)	1 (2.1)	2 (2.1)	1.0
Coronary artery pattern				1.0
Usual	43 (91.5)	43 (91.5)	86 (91.5)	
Atypical pattern	4 (8.5)	4 (8.5)	8 (8.5)	
1L2CxR	3 (6.4)	2 (4.3)	5 (5.3)	
1LCxR	1 (2.1)	1 (2.1)	2 (2.1)	
2LCxR	0	1 (2.1)	1 (1.1)	
Intramural course	3 (6.4)	1 (2.1)	4 (4.3)	0.62
Preoperative septostomy	3 (6.4)	3 (6.4)	6 (6.4)	1.0
Propensity score	0.5±0.1	0.5±0.1	0.5±0.1	0.93

Cx=circumflex coronary artery; IVS=intact ventricular septum; L=left main coronary artery; R=right coronary artery; SD=standard deviation; TGA=transposition of the great arteries; VSD=ventricular septal defect Group 1=patients undergoing modified closed coronary transfer; Group 2=patients undergoing trap-door coronary transfer

**Table 3:** Operative data and postoperative outcomes.

Variables (%, mean±SD, median-interquartile ranges)	Group 1 (n:47)	Group 2 (n:47)	All patients (n:94)	*P*-value
VSD closure method				0.93
by autologous pericardium patch	11 (23.4)	13 (27.7)	24 (25.5)	
by PTFE patch	2 (4.3)	2 (4.3)	4 (4.3)	
primary closure	3 (6.4)	4 (8.5)	7 (7.4)	
No LeCompte maneuver	0	2 (4.3)	2 (2.1)	0.50
Aortic arch reconstruction	3 (6.4)	3 (6.4)	6 (6.4)	1.0
Coarctation repair	1 (2.1)	1 (2.1)	2 (2.1)	1.0
Reintervention for coronary trouble during the operation	1 (2.1)	0	1 (1.1)	1.0
Cross-clamp time (min)	97.3±33.6	115.2±32.3	106.1±33.9	0.03*
CPB time (min)	162.2±49.3	169.5±57.0	165.9±53.0	0.54
Operation time (min)	253.5±57.7	296.7±109.1	273.6±87.7	0.05*
Complication				
Delayed sternal closure	10 (21.7)	12 (25.5)	22 (23.4)	0.67
Arrhythmia	1 (2.1)	1 (2.1)	2 (2.1)	1.0
Infection	3 (6.4)	4 (8.5)	7 (7.4)	0.69
Peritoneal dialysis	4 (8.5)	5 (10.6)	9 (9.6)	0.73
ECMO	3 (6.4)	2 (4.3)	5 (5.3)	0.65
LOS in ICU (day)	7.5 (5-17)	7 (4-13)	7.5 (5-15)	0.19
LOS in hospital (day)	14.5 (10-28)	14 (8-19)	14 (10-22)	0.13
Mortality	4 (8.5)	2 (4.3)	6 (6.4)	0.68
Follow-up (year)	1.7±0.7	2.8±0.8	2.3±0.9	<0.001*

CPB=cardiopulmonary bypass; ECMO=extracorporeal membrane oxygenator; ICU=intensive care unit; LOS=length of stay; PTFE=polytetrafluoroethylene; SD=standard deviation; VSD=ventricular septal defect Group 1=patients undergoing modified closed coronary transfer; Group 2=patients undergoing trap-door coronary transfer

* Statistically significant parameter

**Table 4:** Postoperative echocardiographic data.

Variables (%)	Group 1 (n:47)	Group 2 (n:47)	All patients (n:94)	*P*-value
Neo-aortic valve regurgitation				0.29
Trivial	16 (34.0)	21 (44.7)	37 (39.4)	
Moderate/severe	0	0	0	
Neo-aortic valve stenosis				
Trivial	1 (2.1)	3 (6.4)	4 (4.3)	0.62
Moderate/severe	0	0	0	
Neo-pulmonary valve regurgitation				
Trivial	10 (21.3)	17 (36.2)	27 (28.7)	0.11
Moderate/severe	0	0	0	
Neo-pulmonary stenosis				
Trivial (< 20 mmHg)	10 (21.3)	10 (21.3)	20 (21.3)	1.0
Mild (20-40 mmHg)	15 (31.9)	11 (23.4)	26 (27.7)	0.36
Moderate (41-60 mmHg)	2 (4.3)	2 (4.3)	4 (4.3)	1.0

Group 1=patients undergoing modified closed coronary transfer; Group 2=patients undergoing trap-door coronary transfer

**Fig. 4A:**
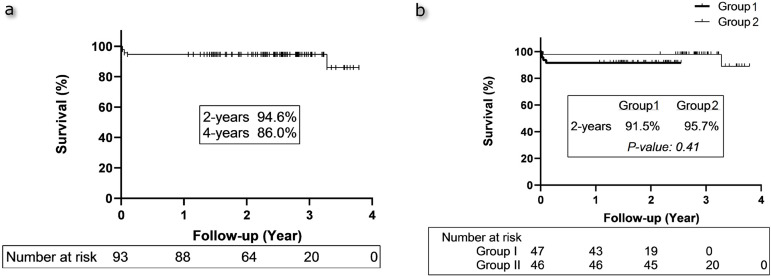
Survival curve for all patients. **Fig. 4B** - Survival curves according to the groups.

In the late period, echocardiographic examination revealed normal ventricular function and normal neo-aortic valve morphology in all patients. In addition to this, five patients had severe neo-pulmonary stenosis (three in group 1, two in group 2, *P*=0.65) at follow-up time. Catheter reintervention for neo-pulmonary stenosis was performed in three patients (two in group 1, one in group 2, *P*=0.56), and two patients (one in group 1, one in group 2, *P*=1.0) underwent surgical reconstruction. Two-year and four-year survival for all patients were 93.6% and 85.1%, respectively (Figure 4A). When compared the long-term survival rates, there was no significant difference between the groups (*P*=0.41, Figure 4B).

## DISCUSSION

This retrospective observational study with a mean of 2.3±0.9 years of follow-up demonstrated that both groups have similar early and long-term postoperative outcomes. However, the arterial cross-clamp time and overall operation time were significantly shorter in the modified closed coronary transfer group compared to the other group. Overall mortality was 6.4% in our series and it was similar to the literature^[16]^.

Proper coronary perfusion is a crucial step during ASO. Coronary perfusion failure is the most common reason of early mortality after ASO^[16]^. Many techniques have been reported related to the coronary artery transfer for both usual coronary pattern^[17]^ and complex coronary pattern^[18-21]^. The most commonly used of these is the trap-door coronary transfer technique, which minimizes the rotation angle of a coronary artery by hinging to the neo-aortic root as trap-door^[12]^. However, it is not adequate for optimal geometric configuration of the transferred coronary arteries^[22]^. Therefore, L Bove et al. described a method, the closed coronary transfer, to achieve that optimal geometric configuration^[23]^. According to this technique, after the neo-aortic reconstruction, the coronary anastomotic zones at the neo-aortic root are adjusted regarding optimal coronary alignment, and the coronary transfer is then applied. The advantage of this procedure is that proper coronary alignment is designed and minimal coronary mobilization is handled, resulting in the optimal coronary artery geometry^[17]^. We performed a modification of this technique in our patients. According to our modification, before the coronary reimplantation to the neo-aortic root, the only posterior half (not all) of anastomosis, between neo-aorta and ascending aorta, is made differently from the technique of L Bove. In this way, the neo-aortic valve can be seen clearly and not be injured while performing coronary artery anastomosis. After the coronary reimplantation, we continue to complete the anastomosis between the neo-aortic root and ascending aorta. The arrangement of coronary geometry is easier in our modified technique and it also reduces time of anastomosis.

Although the classical closed coronary transfer method may be applied to almost all types of coronary pattern, it can be difficult in the presence of side-by-side great artery relationship - Yacoup types b and c of coronary variations^[24]^. Additionally, in the presence of an intramural coronary artery, there is a surgical challenge during ASO. Furthermore, it is associated with a higher mortality than the one from other coronary artery patterns^[25]^. We performed our modified technique to the abnormal coronary pattern in addition to normal pattern. Moreover, there was a side-by-side relationship of the great arteries in a patient in whom was performed our modified technique. Unluckily, in one of three patients with intramural course of coronary artery, we had to perform reimplantation of that coronary button due to coronary perfusion problem during the operation. In contrast, there was no coronary perfusion problem in the other two patients with intramural course.

As another matter, the possible concern about the trap-door coronary transfer method is that there may be a risk for neo-aortic valve regurgitation after ASO. In a study by Formigari et al.^[25]^, it is reported that the trap-door type of coronary reimplantation is associated with an increased risk for valvular disfunction, possibly because of a distortion of the sinotubular junctional geometry^[25]^. Closed coronary transfer technique preserves the sinotubular junction and reduces the risk of late neo-aortic valve regurgitation in this way^[23]^. Similarly, our current study also demonstrates that there was no moderate and/or severe neo-aortic regurgitation in our cohort. Furthermore, the rate of trivial neo-aortic regurgitation is lower in the patients from the modified technique group than in those in whom was performed the trap-door method, even if there was no statistically significance.

On the other hand, the advantage of our modification comparing to the classical closed coronary transfer technique is that there is no concern about injury to the neo-aortic valve while transferring the coronary arteries, because the valve is clearly seen during the coronary reimplantation. However, in the classical closed coronary transfer method, the valve is never in the field of view, resulting in the possible risk of damage to the neo-aortic valve while creating a hole at the neo-aortic root for the coronary reimplantation and performing the coronary artery anastomosis to the neo-aorta. Chang et al.^[17]^ reported that they have performed this method by making a marking stitch at the site of the anterior commissural attachment of the neo-aorta in order to overcome the possible damage to the neo-aortic valve. Choi et al.^[26]^ have also suggested this maneuver in their report. Nonetheless, we do not think this maneuver will eliminate the mentioned risk. Meanwhile, the aortic cross-clamp is removed and replaced during coronary alignment in the classical closed coronary method. This movement of clamp may weaken the aortic wall, which may cause the risk of aneurysm and dissection in the future. In our method, there is no need for this movement of the aortic cross-clamp.

### Limitations

Our study has several limitations. The retrospective design is the major limitation. However, despite the retrospective nature of our study, the patient randomization was provided by using propensity-matching analysis as in a prospective study. Another limitation is that we could not compare the outcomes of our modified technique with the classical closed coronary transfer method. A final limitation is that our study does not include multivariate analysis in order to determine the predictors for mortality because there was not a parameter demonstrating a statistically significant result. Despite these limitations, our study clearly establishes the comparison of two surgical techniques for coronary reimplantation in the early and late periods.

## CONCLUSION

Our modified closed coronary transfer group demonstrates overall good outcomes, comparable to our trap-door method group, and it ensures shorter arterial cross-clamp times and operation times. It is observed less risk of neo-aortic valve regurgitation using this method after ASO. Besides, this modification has an advantage compared to the classical closed coronary transfer technique in terms of preventing the risk of damage to the neo-aortic valve. Thus, it may be an alternative method to the trap-door technique for the coronary transfer during ASO.

**Table:** 

Authors' roles & responsibilities	
MD	Substantial contributions to the conception or design of the work; or the acquisition, analysis, or interpretation of data for the work; drafting the work or revising it critically for important intellectual content; final approval of the version to be published
GC	Drafting the work or revising it critically for important intellectual content
FÖ	Drafting the work or revising it critically for important intellectual content; final approval of the version to be published
OY	Agreement to be accountable for all aspects of the work in ensuring that questions related to the accuracy or integrity of any part of the work are appropriately investigated and resolved
OK	Substantial contributions to the conception or design of the work; or the acquisition, analysis, or interpretation of data for the work
MÇ	Agreement to be accountable for all aspects of the work in ensuring that questions related to the accuracy or integrity of any part of the work are appropriately investigated and resolved
MB	Substantial contributions to the conception or design of the work; or the acquisition, analysis, or interpretation of data for the work
FIC	Agreement to be accountable for all aspects of the work in ensuring that questions related to the accuracy or integrity of any part of the work are appropriately investigated and resolved
NAA	Agreement to be accountable for all aspects of the work in ensuring that questions related to the accuracy or integrity of any part of the work are appropriately investigated and resolved
AS	Agreement to be accountable for all aspects of the work in ensuring that questions related to the accuracy or integrity of any part of the work are appropriately investigated and resolved
